# Neutrophils inhibit γδ T cell functions in the imiquimod-induced mouse model of psoriasis

**DOI:** 10.3389/fimmu.2022.1049079

**Published:** 2022-11-15

**Authors:** Sara Costa, Dalila Bevilacqua, Elena Caveggion, Sara Gasperini, Elena Zenaro, Francesca Pettinella, Marta Donini, Stefano Dusi, Gabriela Constantin, Silvia Lonardi, William Vermi, Francesco De Sanctis, Stefano Ugel, Tiziana Cestari, Clare L. Abram, Clifford A. Lowell, Pamela Rodegher, Franco Tagliaro, Giampiero Girolomoni, Marco A. Cassatella, Patrizia Scapini

**Affiliations:** ^1^ Department of Medicine, Division of General Pathology, University of Verona, Verona, Italy; ^2^ Department of Molecular and Translational Medicine, University of Brescia, Brescia, Italy; ^3^ Division of Immunology, University of Verona, Verona, Italy; ^4^ Department of Laboratory Medicine, University of California, San Francisco, San Francisco, CA, United States; ^5^ Department of Diagnostics and Public Health, Unit of Forensic Medicine, University of Verona, Verona, Italy; ^6^ Division of Dermatology and Venereology, University of Verona, Verona, Italy

**Keywords:** neutrophils, gamma delta (gammadelta) T cells, skin inflammation, inflammatory cyotokines, immunoregulation

## Abstract

**Background:**

Psoriasis is a chronic skin disease associated with deregulated interplays between immune cells and keratinocytes. Neutrophil accumulation in the skin is a histological feature that characterizes psoriasis. However, the role of neutrophils in psoriasis onset and development remains poorly understood.

**Methods:**

In this study, we utilized the model of psoriasiform dermatitis, caused by the repeated topical application of an imiquimod containing cream, in neutrophil-depleted mice or in mice carrying impairment in neutrophil functions, including p47phox -/- mice (lacking a cytosolic subunit of the phagocyte nicotinamide adenine dinucleotide phosphate - NADPH - oxidase) and Sykfl/fl MRP8-cre+ mice (carrying the specific deletion of the Syk kinase in neutrophils only), to elucidate the specific contribution of neutrophils to psoriasis development.

**Results:**

By analyzing disease development/progression in neutrophil-depleted mice, we now report that neutrophils act as negative modulators of disease propagation and exacerbation by inhibiting gammadelta T cell effector functions via nicotinamide adenine dinucleotide phosphate (NADPH) oxidase-mediated reactive oxygen species (ROS) production. We also report that Syk functions as a crucial molecule in determining the outcome of neutrophil and γδ T cell interactions. Accordingly, we uncover that a selective impairment of Syk-dependent signaling in neutrophils is sufficient to reproduce the enhancement of skin inflammation and γδ T cell infiltration observed in neutrophil-depleted mice.

**Conclusions:**

Overall, our findings add new insights into the specific contribution of neutrophils to disease progression in the IMQ-induced mouse model of psoriasis, namely as negative regulatory cells.

## Introduction

Psoriasis has for a long time been considered a skin disease primarily based on disturbances of epidermal homeostasis (1). However, it is currently clear that at the basis of its pathogenesis there are complex interplays between keratinocytes and immune cells that are in turn influenced by psoriasis-associated susceptibility loci, autoantigens, and multiple environmental factors (2–4). Deregulated axis involving the overproduction of interleukin 23 (IL-23), and the consequent activation of IL-17-producing T cell subsets (T17), recently emerged as the central immune pathway driving the development of psoriasis (5, 6). Also, the overproduction of other inflammatory cytokines, such as IL-1, IL-36, TNFα, and IL-22, is known to trigger pivotal pathogenic pathways in human psoriasis (2–4). Among the cellular mediators, besides T17 - which include T helper 17 (Th17) and γδ T cells) (7–9), also the crucial role of DCs (10, 11) has been widely studied in human psoriasis and its preclinical models. By contrast, the role of myeloid cells (such as neutrophils, monocytes, and macrophages), which are also known to infiltrate the psoriatic plaques and to display abnormal functions in psoriatic patients, in disease pathogenesis is less well-characterized (2, 12, 13).

Neutrophils are the most abundant leukocytes in humans and play a pivotal role in driving defensive responses toward various infection types (14–16). Recently, it has become clear that the functions of neutrophils go far beyond the elimination of microorganisms and that these cells may contribute to the pathogenesis of numerous chronic inflammatory disorders (16–18). In this context, the presence and infiltration of neutrophils into the epidermis is one of the histologic hallmarks of psoriasis (1, 2). The most credited hypothesis view neutrophils as the principal cellular mediators in the IL-17–dependent pathophysiology of psoriasis, suggesting a proinflammatory role of neutrophils in this disease (13, 19, 20). However, emerging data from clinical evidence do not allow drawing definitive conclusions. Indeed, while early clinical findings reported that agranulocytosis can improve the outcome in patients with different subtypes of psoriasis (21, 22), more recent clinical trials aimed at interfering with neutrophil recruitment or functions into the inflammatory skin (e.g. anti-human CXCL8 Abs) were not successful (13, 23). Similar controversial results on the pathogenic role of neutrophils in psoriasis also emerge from studies in which preclinical models of this disease have been utilized (19, 24–26).

To better elucidate the role of neutrophils in psoriasis development, we have utilized the imiquimod (IMQ)-induced mouse model of psoriasis, which consists of the topical administration of Aldara ™ cream - containing the Toll-like receptor 7 and 8 (TLR7/8) ligand IMQ (5%) (3, 27). This model is broadly utilized to elucidate pathogenic mechanisms involved in psoriasis development as well as to evaluate possible new therapies for this disease (3, 27–29). While dendritic cells (DCs) and T cells (mostly γδ T cells) are thought to be crucial to the pathogenesis of IMQ-induced psoriasis (24, 30–34), the role of neutrophils in this model remains unclear. Indeed, neutrophil depletion resulted in a reduction of IMQ-induced psoriasis in two studies (25, 26), or did not affect disease development in another study (24).

Herein, by performing neutrophil depletion or utilizing mice carrying impairment in neutrophil functions, including *p47^phox^* ^-/-^ mice [lacking a cytosolic subunit of the phagocyte NADPH oxidase (35)] and *Syk^fl/fl^MRP8-cre^+^* mice [carrying the specific deletion of the Syk kinase in neutrophils only (36, 37)] we uncover a novel potential regulatory role of neutrophils in IMQ-induced psoriasis.

## Materials and methods

### Mice

*Syk^fl/fl^* and *Syk^fl/fl^Mrp8-cre^+^* mice, were previously described (Van Ziffle & Lowell 2009), *p47phox^−/−^* mice were a gift from Prof. Romani (University of Perugia) and were previously described (35). *Tcrb*_-/-_ mice were a gift from Prof. Constantin (University of Verona). C57BL/6 mice were purchased from The Jackson Laboratory (Bar Harbor, ME, USA). All mice used in this study were on a C57BL/6 background and kept in a specific pathogen-free facility.

### IMQ-induced psoriasis model

For induction of psoriasis-like skin inflammation, mice at 8–12 wk of age received a daily topical dose of 62,5 mg of commercially available IMQ cream (5%) (Aldara Cream™, Meda AB) or control cream (vaseline) on their shaved backs for 6 consecutive days as previously described (28, 38). On the fourth or seventh day, the animals were euthanized. Back skin was isolated, and half was fixed in 10% formaldehyde for histopathology analysis while the other half was finely chopped and stored in RNAlater (Ambion) for quantitative real-time PCR (qRT-PCR) or digested, as described below, to achieve single-cell suspensions for flow cytometry analysis.

### Neutrophil depletion

Mice were injected intra-peritoneally (i.p.) with 300 μg of rat anti-mouse Ly6G Ab (clone 1A8; BioXcell) or isotype control Rat IgG2a (clone 2A3; BioXCell), dissolved in 300 ul phosphate-buffered saline (PBS) every other day from day 0 to day 6.

### Cell preparation and flow cytometry

Skin tissue (2 cm X 2 cm) was cut from dorsal skin of the mouse. After removing subcutaneous tissue and collagen intensively with forceps, the skin was cut into small pieces and digested with 0,4 mg/ml Liberase TM (Roche Ltd.) and 0,5 mg/ml DNase I (Sigma) in RPMI 1640 medium (Sigma) for 1 hour. Single cell suspension was obtained by shredding with gentle Macs Dissociator (Miltenyi Biotec) and filtering with 70 μm and 40 μm cell strainer in series. Lymph nodes were mechanically dissociated by two frosted microscope slides and passage through a 70 μM cell strainer to yield a single-cell solution. Cells were resuspended in phosphate buffered saline containing 2% (vol/vol) fetal calf serum, 2 mM EDTA and maintained at 4°C. For flow cytometry, 1–2×10^6^ cells were stained. Non-specific binding was blocked by pre-incubation with 0.5 µg anti-CD16/32 (2.4G2, Biolegend) and 100 µg mouse IgG (Sigma). Surface staining was performed with the following anti-mouse Abs: Ly6G(1A8), TCRαβ (H57-597), CD62L (MEL-14), CD11b (M1/70), CD45 (30-F11), I-Ab (MHCII)(AF6-120.1), CD44 (IM7), TCR γ/δ (GL3) from Biolegends; Ly6C (AL-21), CD11c (HL3), CD3 (145-2C11) and GR-1 (RB6-8C5), from BD Biosciences. After final wash, cells were resuspended in staining/wash buffer containing 1 mg/ml propidium iodide (PI; Sigma-Aldrich) for viability staining according to the manufacturer’s instructions. For intracellular cytokine staining, the cells were activated for 4 hours in phorbol 12-myristate 13-acetate (PMA; 50 ng/ml) and ionomycin (750 ng/ml) in the presence of brefeldin A (1 mg/ml). Thereafter, cells were surface-stained, washed, and then fixed and permeabilized using the eBioscience kit as previously described (39). Intracellular staining was performed with anti-mouse IL-17A (TC11-18H10.1; eBioscience) or its relevant isotype control mAbs. Sample fluorescence was measured by a seven-color MACSQuant Analyzer (Miltenyi Biotec), while data analysis was performed by using FlowJo software Version 8.8.6 (Tree Star, Ashland, OR, USA).

### Quantitative real-time PCR

Real-time reverse transcription-PCR was performed, as previously described (40), using total RNA isolated from 30 mg of the skin by RNeasy Fibrous Tissue Mini Kit (QIAGEN) and utilizing the following gene-specific primer pairs (all purchased from Invitrogen) (**Supplementary Table 1**). Data were calculated by Q-Gene software (http://www.gene **)** quantification.de/download.html) and expressed as mean normalized expression (MNE) units after RPL32 normalization.

### Skin histology and immunohistochemistry

Dorsal skin samples (3 mm) were obtained by a transversal cut of the central skin area, fixed in 10% neutral buffered formalin and embedded in paraffin blocks by using a Tissue-Tek^®^ Tissue Embedding Console System from Diapath (Bergamo, Italy). The paraffin blocks were cut into 3 µm thick cross-sections and stained with hematoxylin and eosin following the standard procedure (immersion in Mayer’s hematoxylin: 2 minutes; immersion in eosin: 3 min) by using a Leica Microsystem Autostainer XL ST5010 (Milano, Italy). Epidermal thickness was determined by measuring the average interfollicular distance under the microscope in a blinded manner. Pictures were taken using Leica DFC 300FX Digital Color Camera on a Leica DM 6000 B microscope at a 100x magnification. For γδ T cell and neutrophil immunohistochemical staining, 4 µm formalin-fixed, paraffin-embedded tissue sections were double stained after appropriate antigen retrieval with rat anti-mouse RORgt (dilution 1:50, clone AFKJS-9, eBiosciences, San Diego, CA, USA) and Ly6G (dilution 1:400, clone 7/4, Cedarlane, Burlington, On, Canada).The first immune reaction was revealed using rat-on-mouse HRP-Polymer (Biocare Medical, Concord, CA, USA) and developed by diaminobenzidine; the slides were then incubated with anti-Ly6G, revealed using rat-on-mouse AP-Polymer and developed with vector Blue chromogen (Vector Laboratories, Newark, CA, USA). Slides were then counterstained with hematoxylin. Slides were photographed using the DP73 Olympus digital camera mounted on an Olympus BX60 microscope and resized using Adobe Photoshop.

### Statistical analysis

Data were expressed as the mean ± SD and analyzed using GraphPad Prism Version 5 software (GraphPad Software, Inc.). The comparison of variables was performed using two-tailed Student *t*- test (for comparison between two groups) or a 1-way ANOVA with Bonferroni’s posttest (used for multiple comparisons), Dunnett’s post-test (when multiple comparisons to control group were made). P-values of less than 0.05 were considered significant and symbols indicate significant increases: *^/#^, *P <*0.05; **^/##^, *P* ≤ 0.01; ***^/###^, *P* ≤ 0.001; ^****/####^, P ≤ 0.0001. Graphs were elaborated using GraphPad Prism Version 5 software (GraphPad Software, Inc.).

### Online supplementary material

This includes extended methods, one Table and four Figures.

## Results

### Neutrophil depletion reduces the progression, but not the initiation, of skin inflammation and epidermal thickening in the IMQ-induced mouse model of psoriasis

**Figure 1:**
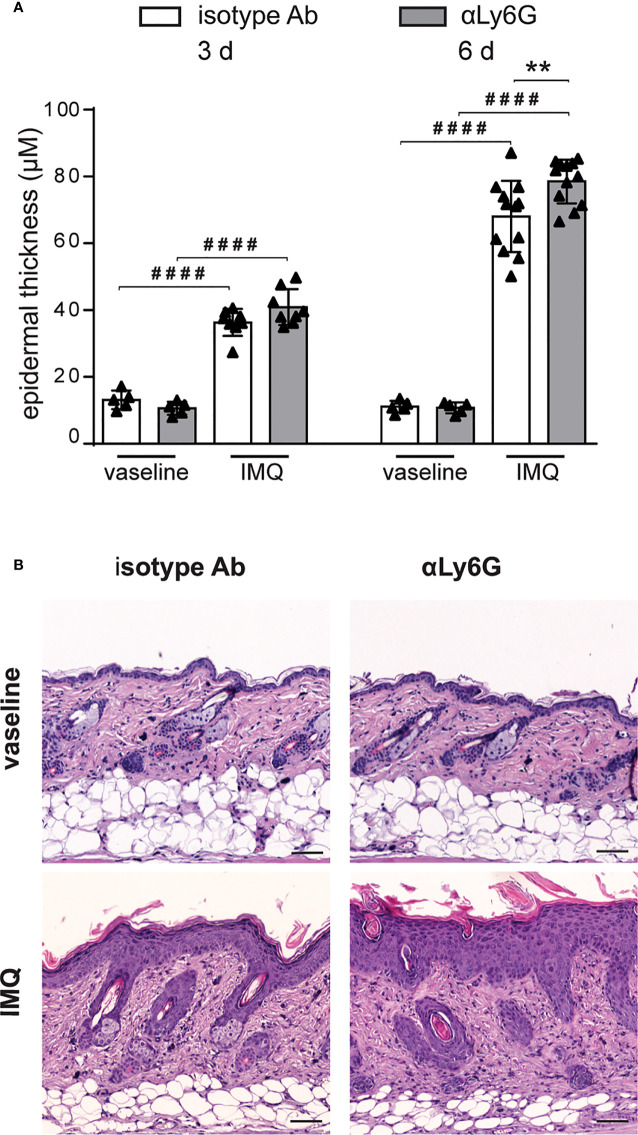
Increased epidermal thickening in neutrophil-depleted mice in response to IMQ treatment. Dorsal skin of mice was topically treated with vaseline or IMQ-containing cream (Aldara^®^) for 3 or 6 consecutive days. Mice were injected with the depleting antibody αLy6G or isotype control antibody. **(A)** The height of epidermal hyperplasia was measured in interfollicular epidermis on H&E-stained slides by light microscopic evaluation. Data are pooled from 3 separate time course experiments and are expressed as means ± SD (n = 5-12). Statistical differences of IMQ-treated *vs*. vaseline-treated mice (#) and IMQ-treated control *vs*. neutrophil-depleted mice (*) are reported. **P ≤ 0.01; ####*P* ≤ 0.0001 by 1-way ANOVA with Bonferroni’s post-test. **(B)** Representative H&E-staining of dorsal skin from mice injected with isotype Ab or αLy6G treated with vaseline or IMQ for 6 days. Original magnification, X100; original scale bars 40μm.

**Figure 2:**
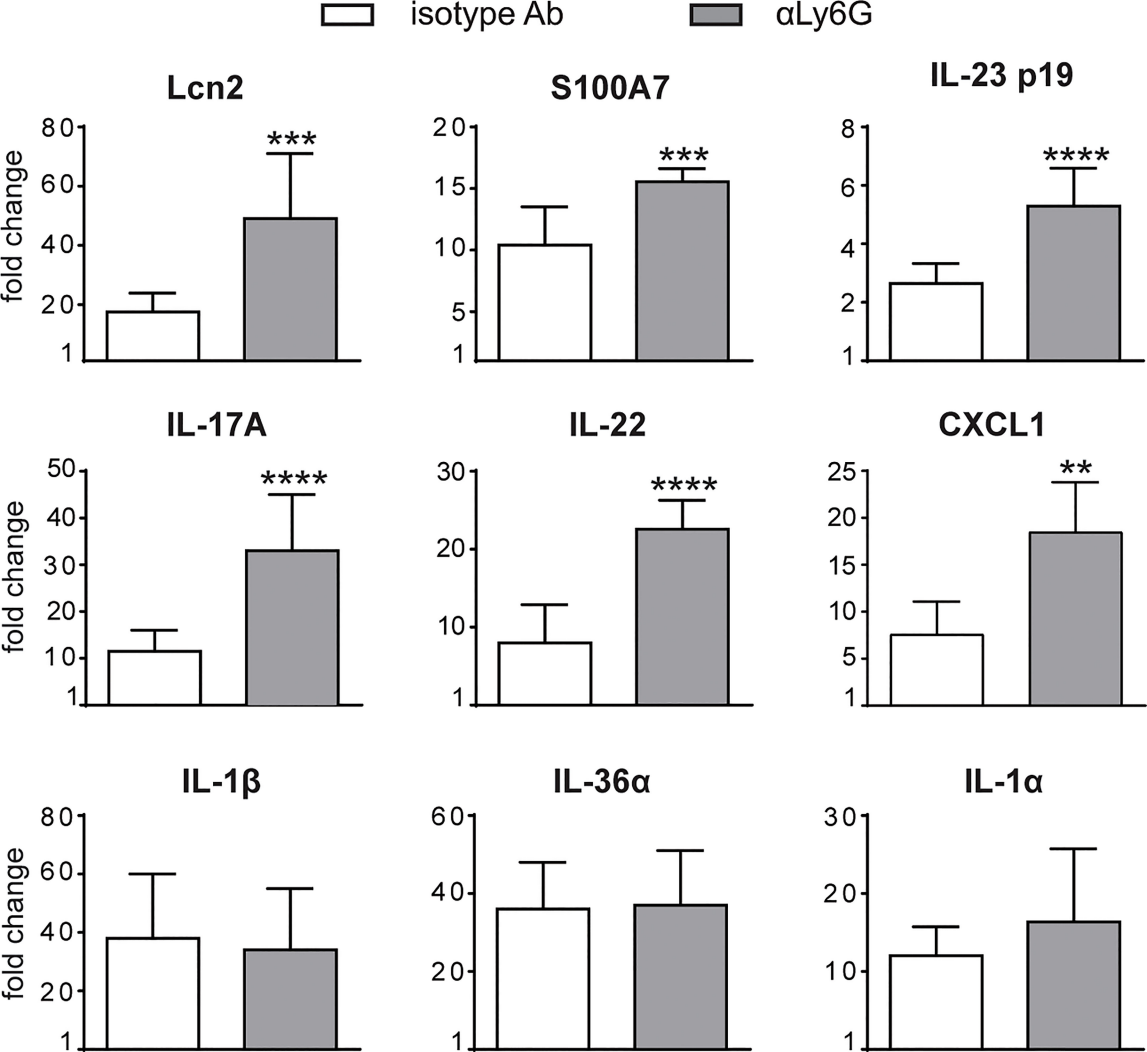
Gene-expression analysis of inflammatory molecules in the skin of IMQ-treated control or neutrophil-depleted mice. The dorsal skin of mice was topically treated with IMQ-containing cream (Aldara^®^) or vaseline for 6 consecutive days. Mice were injected with the depleting antibody αLy6G or isotype control Ab. Total skin RNA was extracted and reverse transcribed. mRNA expression of the indicated genes for IMQ-treated control or neutrophil-depleted mice is displayed as fold change of MNE units (after RPL32 normalization) over vaseline-treated control. Data are pooled from 2 separate experiments and are expressed as means ± SD (n = 8-12 mice). Statistical differences of IMQ-treated control *vs*. neutrophil-depleted mice (*) are reported. ***P ≤ 0.001; **P ≤ 0.01; ****P ≤ 0.0001 by *t-*test.

To investigate the specific contribution of neutrophils to the development of IMQ-induced psoriasis, we performed neutrophil depletion by injecting anti-Ly6G (clone 1A8) Ab, or isotype control Ab, in mice treated with IMQ (or vaseline control cream), for 3 or 6 consecutive days as originally described by Vanderfits et al. (28). First, we confirmed that the anti-Ly6G–treatment successfully depleted neutrophils in lymph nodes and the skin of either vaseline or IMQ-treated mice after both 3 and 6 days of treatment (**Supplementary Figures 1A–C**). Interestingly, neutrophil depletion did not significantly affect epidermal thickening, the most utilized and reproducible clinical parameter utilized to quantify disease severity in this model (27), up to 3 days of IMQ treatment (**Figure 1A**). However, differently from what was previously published by others (24, 25), we observed an unexpected significant increase of epidermal thickening in neutrophil-depleted mice, as compared to control mice, upon 6 days of IMQ treatment (**Figures 1A, B**). Consistently, the expression of skin-associated psoriatic genes by qRT-PCR, such as Lipocalin-2 (Lcn2) and S100 calcium binding protein A7/psoriasin (S100A7) was significantly higher in dorsal skin of mice IMQ-treated receiving anti-Ly6G Ab, as compared to control IgG-treated mice (**Figure 2**). Strikingly, we also observed that, upon IMQ treatment, mice devoid of neutrophils manifested a significantly increased expression of cytokines implicated in the IL-23/T17 axis, including IL-23, IL-17, IL-22, CXCL1 and IL-6, as compared to control IgG-treated mice (**Figure 2** and **data not shown**). Neutrophil depletion, instead, did not significantly affect the expression of other inflammatory cytokines induced by IMQ treatment, such as IL-1β, IL-36 and IL-1α (**Figure 2**).

Overall, these data suggest a novel potential role for neutrophils as negative modulators of disease progression and of the IL-23/T17 axis in IMQ-induced psoriasis.

### Neutrophil depletion increases the expansion and infiltration of T cells in lymph nodes and skin of IMQ-treated mice

**Figure 3:**
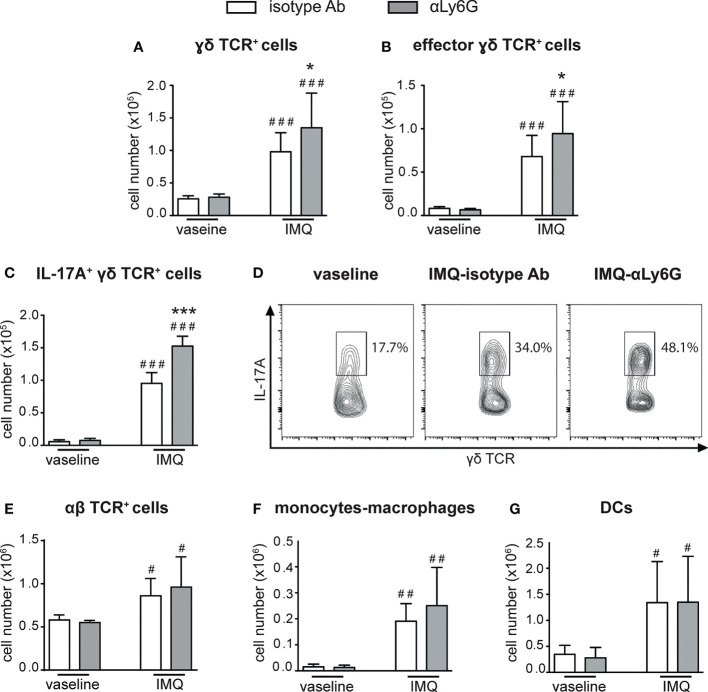
Infiltration of inflammatory cells in the draining lymph nodes of IMQ-treated control or neutrophil-depleted mice. The dorsal skin of mice was topically treated with IMQ-containing cream (Aldara^®^) or vaseline for 6 consecutive days. Mice were injected with the depleting antibody αLy6G or isotype control Ab. Draining lymph nodes were collected and analyzed by flow cytometry. Panels report: the number of total **(A)** and effector (CD44^high^CD62L^low^, **B**) γδ TCR^+^ cells; the total number **(C)** or the frequencies (representative FACS plots**, D**) of IL-17A-producing γδ TCR^+^ cells; the total number of αβ TCR^+^ T cells **(E)**; the total number of monocytes/Mϕ (CD11b^high^Ly6G^-^CD11c^low/-^MHCII^low/-^ cells *plus* CD11b^high^Ly6G**^-^** CD11c^low^/^-^MHCII^high^ cells) **(F)**; the total number of DCs (CD11c^+/high^MHCII^high^) **(G)**. Data are pooled from 3 separate experiments and are expressed as means ± SD (n = 14-15 mice). Statistical differences of IMQ-treated *vs*. vaseline-treated mice (#) and IMQ-treated control *vs*. neutrophil-depleted mice (*) are reported. #/*P ≤ 0.05; ##*P* ≤ 0.01; ###/***P ≤ 0.001 by 1-way ANOVA with Bonferroni’s post-test.

We then performed a careful characterization of the CD45^+^ cells infiltrating the draining lymph nodes and the skin of IMQ-treated mice receiving anti-Ly6G Ab, or control IgG, by flow cytometry, utilizing the gating strategies previously described (38). Interestingly, we found that neutrophil-depleted mice displayed a strongly increased accumulation of γδ T cells in the draining lymph nodes after 6 days of IMQ treatment (**Figure 3A**). Besides the total number, also the number of CD44^high^CD62L^low^ effector γδ T cells (**Figure 3B**) and of IL-17-producing γδ T cells (**Figures 3 C, D**) were significantly increased, indicating that not only the numbers but also the activation state of these cells was profoundly affected by the depletion of neutrophils. No significant differences in the infiltration of αβ T cells (**Figure 3E**), monocytes/macrophages (**Figure 3F**) and DCs (**Figure 3G**) were instead found in the draining lymph nodes of anti-Ly6G–treated mice when compared to controls.

**Figure 4:**
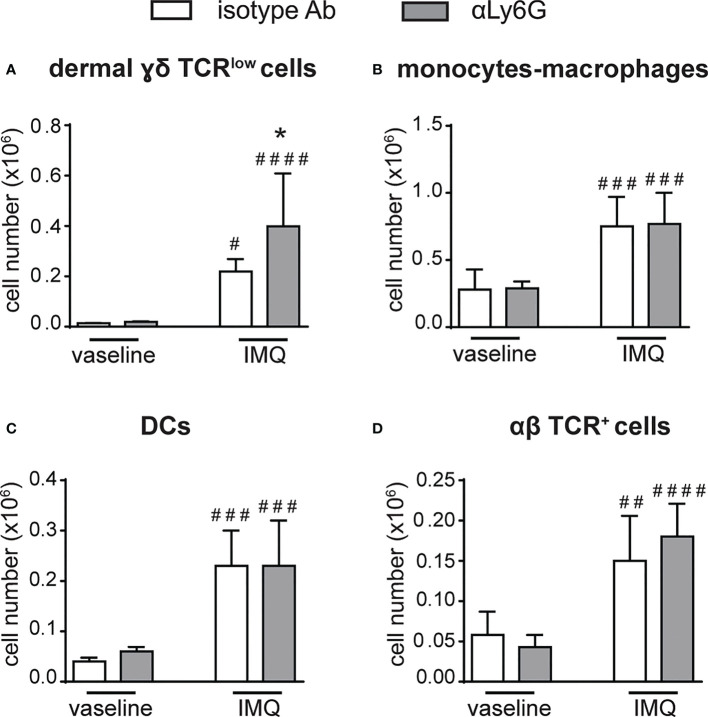
The infiltration of γδ T cells is increased in the skin of neutrophil-depleted mice treated with IMQ. The dorsal skin of mice was topically treated with IMQ-containing cream (Aldara^®^) or vaseline for 6 consecutive days. Mice were injected with the depleting antibody αLy6G or isotype control Ab. Total skin (2x2 cm) was digested and analyzed by flow cytometry. Panels report the total number of: dermal γδ TCR^low^ T cells **(A)**; monocytes/Mϕ (CD11b^high^Ly6G^-^CD11c^low/-^MHCII^low/-^ cells *plus* CD11b^high^Ly6G**^-^** CD11c^low^/^-^MHCII^high^ cells) **(B)**; DCs (CD11c^+/high^MHCII^high^) **(C)**; αβ TCR^+^ T cells **(D)**. Data are pooled from 2 separate experiments and are expressed as means ± SD (n = 8-10 mice). Statistical differences of IMQ-treated *vs*. vaseline-treated mice (#) and IMQ-treated control *vs*. neutrophil-depleted mice (*) are reported. #/*P ≤ 0.05; ##*P* ≤ 0.01; ###*P* ≤ 0.001; ####*P* ≤ 0.0001 by 1-way ANOVA with Bonferroni’s post-test.

Notably, a strong expansion of dermal γδ TCR^low^ T cells (**Figure 4A**), but not of monocytes/macrophages, DCs or αβ T cells (**Figures 4B–D**), was also evident in the dorsal skin of anti-Ly6G-treated, as compared to control IgG-treated, mice after 6 days of IMQ treatment. It is worth pointing out that, under our experimental conditions, γδ T cells and neutrophils infiltrated the lymph nodes and the skin of IMQ-treated mice with similar kinetics, and that the infiltration of both cell types appeared much more consistent after 6 rather than 3 days of IMQ-treatment (**Supplementary Figures 1, 2**). Interestingly, γδ T cells and neutrophils infiltrating the skin dermis of IMQ-treated mice appear to be in close contact (**Supplementary Figure 3**). Collectively, our findings suggest neutrophils’ potential negative regulatory role toward the infiltration and expansion of γδ T cells in both the lymph nodes and skin of IMQ-treated mice.

### Neutrophils inhibit the proliferation and the production of IL-17 by γ T cells *via* ROS production

**Figure 5:**
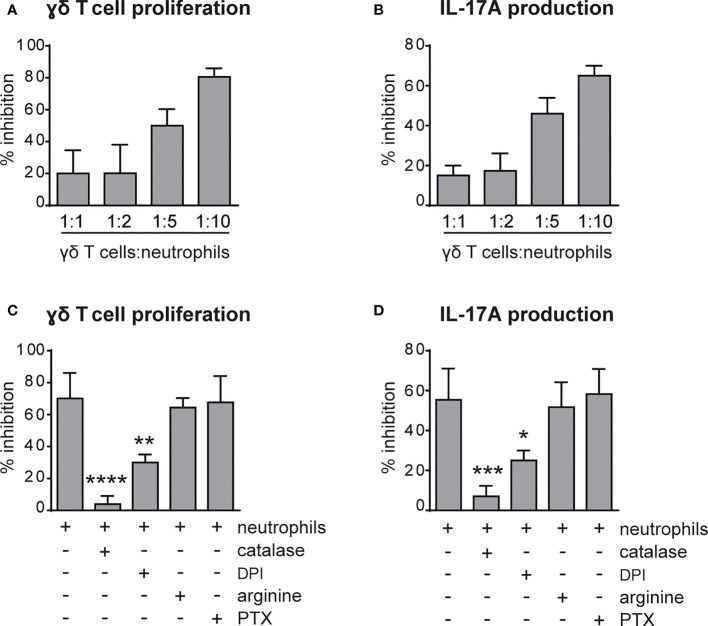
Neutrophils inhibit the proliferation and IL-17 production by γδ T cells *via* reactive oxygen species (ROS) production. **(A, B)** γδ T cells were stimulated with CD3/CD28, 100 ng/ml IL-23 *plus* 10 ng/ml IL-1β and cultured for 72h in the presence or absence of neutrophils at different ratios. **(C, D)** γδ T cells were stimulated with CD3/CD28, 100 ng/ml IL-23 *plus* 10 ng/ml IL-1β and cultured for 72h with neutrophils added at a 1 to 5 γδ T to neutrophil cell ratio, with or without inhibitors: catalase (1000 U/ml), diphenyleneiodonium (DPI) (0,1 μM), L-arginine (200 μg/ml-1), pentoxifillin (PTX) (0,5 μM). The percentages of inhibition of proliferation, as measured by BrdU incorporation **(A, C)**, or IL-17A production **(B, D)** by γδ T cells, are reported. Graph values indicate means ± SD from 2 to 3 independent experiments. Statistical differences of the effect of neutrophils in the presence or absence of inhibitors are reported. *P ≤ 0.05; **P ≤ 0.01; ***P ≤ 0.001; ****P ≤ 0.0001 by 1-way ANOVA with Dunnett’s post-test.

**Figure 6:**
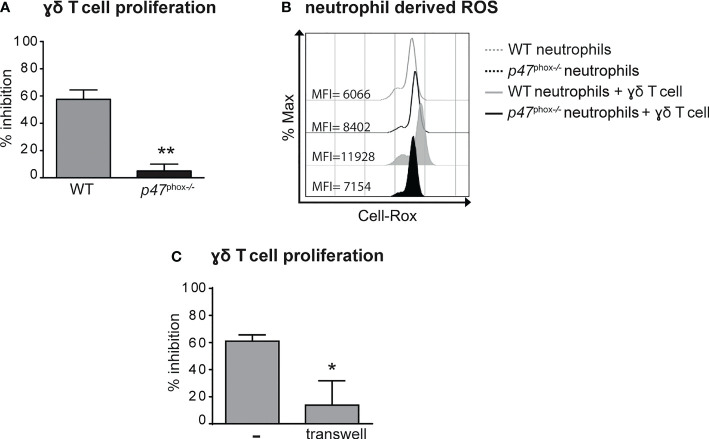
Neutrophil-mediated inhibition of γδ T cell proliferation requires (NADPH) oxidase-dependent ROS production and direct cell-to-cell contacts. γδ T cells were stimulated with CD3/CD28, 100 ng/ml IL-23 *plus* 10 ng/ml IL-1β and cultured with neutrophils from either wild-type (WT) or *p47^phox-/-^* mice for 72 **(A, C)** or 3 **(B)** hours. **(A)** Percentages of inhibition of γδ T cell proliferation by neutrophils from WT or *p47^phox-/-^* mice as measured by BrdU incorporation.**(B)** Representative FACS histogram plots depicting the Cell-RoX MFI of CD11b^+^Ly6G^+^ neutrophils from WT or *p47^phox-/-^* mice in the presence or absence of γδ T cells, as evaluated by FACS analysis. **(C)** Stimulated γδ T cells were cultured with neutrophils under direct contact or transwell conditions. The graph shows the percentages of inhibition of γδ T cell proliferation, as measured by BrdU incorporation. Graph values indicate means ± SD from 2 independent experiments. *P ≤ 0.05; **P ≤ 0.01, by *t-*test.

Previous findings have highlighted the capacity of neutrophils to both positively and negatively modulate the effector functions of γδ T cells (41–43). Therefore, we tested the immunomodulatory roles of neutrophils on the proliferation and the production of IL-17 by γδ T cells stimulated *in vitro* with plate-bound anti-CD3 Abs and soluble anti-CD28 Abs in the presence of 100 ng/mL IL-23 and ˜1 ng/mL IL-1β, as previously described (31, 38). As shown in **Figures 5A, B**, neutrophils inhibited both the proliferation and the production of IL-17, respectively, by activated γδ T cells. Given that the degree of this inhibitory effect was ratio-dependent (**Figures 5A, B**), in all subsequent experiments we used the 5/1 neutrophil/T cell ratio, a condition in which we obtained a strong and reproducible inhibition of γδ T cell functions by neutrophils. In agreement with previous studies (42, 44), we found that the addition of either catalase (a H_2_O_2_ scavenger) or of diphenyleneiodonium (DPI, a NADPH oxidase inhibitor) almost completely reverted the immunosuppressive functions of mouse neutrophils on γδ T cells (**Figures 5C, D**). Other inhibitors of neutrophil’s effector functions such as pentoxyfilline (PTX, a degranulation inhibitor) or L-arginine [an arginase-1 (ARG1) inhibitor] were effective neither on the proliferation nor on the production of IL-17 in activated γδ T cells (**Figures 5C, D**). In line with these observations, neutrophils isolated from *p47^phox^* ^-/-^ mice, that lack NOX2 activity, were unable to effectively inhibit γδ T cell proliferation *in vitro* (**Figure 6A**). Consistently, by performing a flow cytometric measurement of ROS production, we also observed that wild-type (WT) neutrophils, but of not *p47^phox-/-^* neutrophils, produce ROS in the presence of γδ T cells in the culture (**Figure 6B**). Finally, in line with the fact that the inhibitory functions of different immunosuppressive neutrophil populations have been shown to occur through direct cell contact-dependent mechanisms (45–48), we found that the capacity of neutrophils to inhibit γδ T cell proliferation was significantly lower if neutrophils were physically separated from T cells by the use of transwells (**Figure 6C**).

Taken together, data suggest that neutrophils inhibit γδ T cell functions *via* a cell contact-dependent ROS production.

### Syk signaling modulates the capacity of neutrophils to inhibit T cell functions and disease progression in the IMQ-mouse model of psoriasis

**Figure 7:**
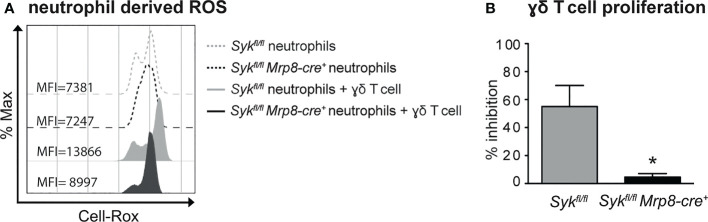
ROS-mediated inhibition of γδ T cell proliferation by neutrophils requires the activation of Syk-dependent signaling pathways. γδ T cells were stimulated with CD3/CD28, 100 ng/ml IL-23 *plus* 10 ng/ml IL-1β and cultured with neutrophils from *Syk^fl/fl^* or *Syk^fl/fl^Mrp8-cre^+^* mice for 3 **(A)** or 72 **(B)** hours. **(A)** Representative FACS histogram plots depicting the CellROX MFI of CD11b^+^Ly6G^+^ neutrophils from *Syk^fl/fl^* or *Syk^fl/fl^Mrp8-cre^+^* mice in the presence or absence of γδ T cells, as evaluated by FACS analysis. **(B)** Percentages of inhibition of γδ T cell proliferation by neutrophils from *Syk^fl/fl^* or *Syk^fl/fl^Mrp8-cre^+^* mice, as measured by BrdU incorporation. Graph values indicate means ± SD from 2 independent experiments. *P ≤ 0.05 by *t*-test.

Spleen tyrosine kinase (Syk), a member of nonreceptor tyrosine kinases, transmits signals in neutrophils from a variety of immunereceptors, including Fcγ receptors (FcγRs) and adhesion molecules, such as β2 integrins and P-Selectin glycoprotein ligand 1 (PSGL-1) (49–51). As a consequence, *Syk ^-/-^* neutrophils display impaired effector functions, including the production of ROS and the release of granule contents, in response to several inflammatory stimuli (50, 51). Syk-based signaling in neutrophils alone was previously shown to be critical for appropriate host defense to *Staphylococcus aureus* (37) or the development of inflammatory arthritis (36), suggesting the relevance of this signaling pathway in neutrophils during immune responses. Therefore, we decided to utilize mice carrying the specific deletion of Syk in neutrophils [*Syk^fl/fl^Mrp8-cre^+^* mice (36, 37),], available in our laboratory, as an experimental model to test whether the specific impairment of this signaling pathway in neutrophils was sufficient to affect their interactions with γδ T cells in IMQ-induced psoriasis. Consistently, *Syk ^-/-^* neutrophils failed to produce ROS and to inhibit the proliferation of γδ T cells in our *in vitro* experimental conditions (**Figures 7A, B**). These data validated therefore Syk as a crucial signaling molecule involved in the modulation of the neutrophil capability to inhibit γδ T cell proliferation *via* a contact-dependent ROS production.

**Figure 8:**
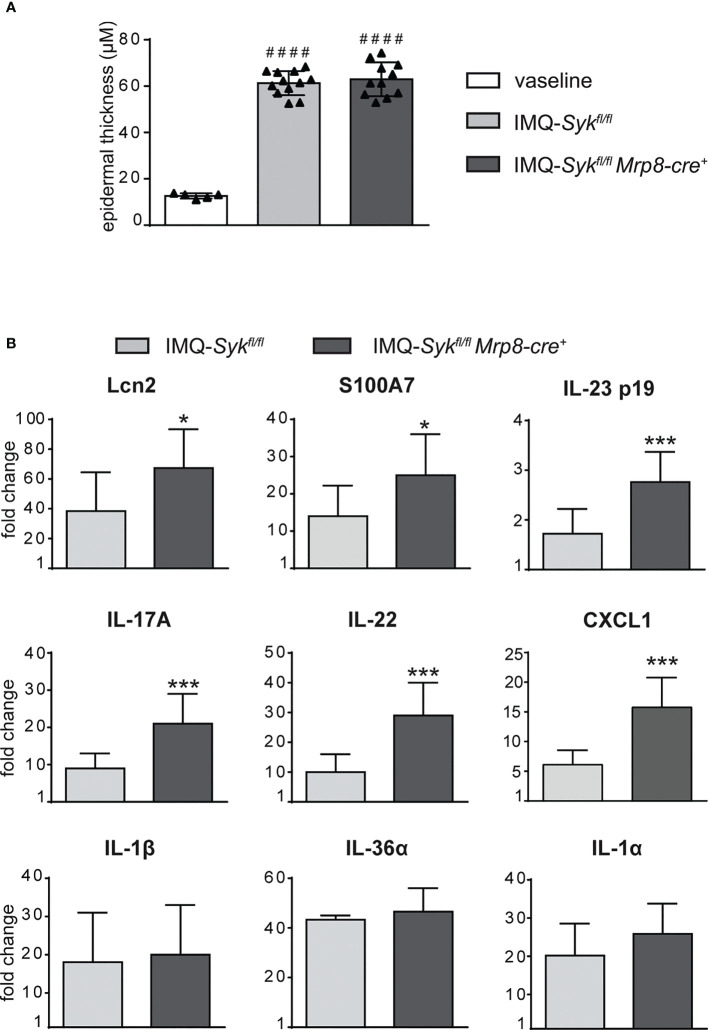
Epidermal thickening and gene-expression analysis of inflammatory molecules in the skin of IMQ-treated *Syk^fl/fl^* and *Syk^fl/fl^Mrp8-cre^+^* mice. The dorsal skin of control *Syk^fl/fl^* and *Syk^fl/fl^Mrp8-cre^+^* mice was topically treated with IMQ-containing cream (Aldara^®^) or Vaseline for 6 consecutive days. **(A)** The height of epidermal hyperplasia (epidermal thickening) was measured in the interfollicular epidermis on H&E-stained slides by light microscopic evaluation. **(B)** Total skin RNA was extracted and reverse transcribed. mRNA expression of the indicated genes for IMQ-treated *Syk^fl/fl^* and *Syk^fl/fl^Mrp8-cre^+^* mice is displayed as fold change of MNE units (after RPL32 normalization) over vaseline-treated controls. Data are pooled from 2 separate experiments and are expressed as means ± SD (n = 11 mice). Statistical differences of IMQ-treated *Syk^fl/fl^* or *Syk^fl/fl^Mrp8-cre^+^* mice *vs*. vaseline-treated mice (#) and IMQ-treated *Syk^fl/fl^ vs*. IMQ-treated *Syk^fl/fl^Mrp8-cre^+^* mice (*) are reported. ####*P* ≤ 0.0001 by 1-way ANOVA with Bonferroni’s post-test. *P ≤ 0.05; ***P ≤ 0.001 by *t-*test.

**Figure 9:**
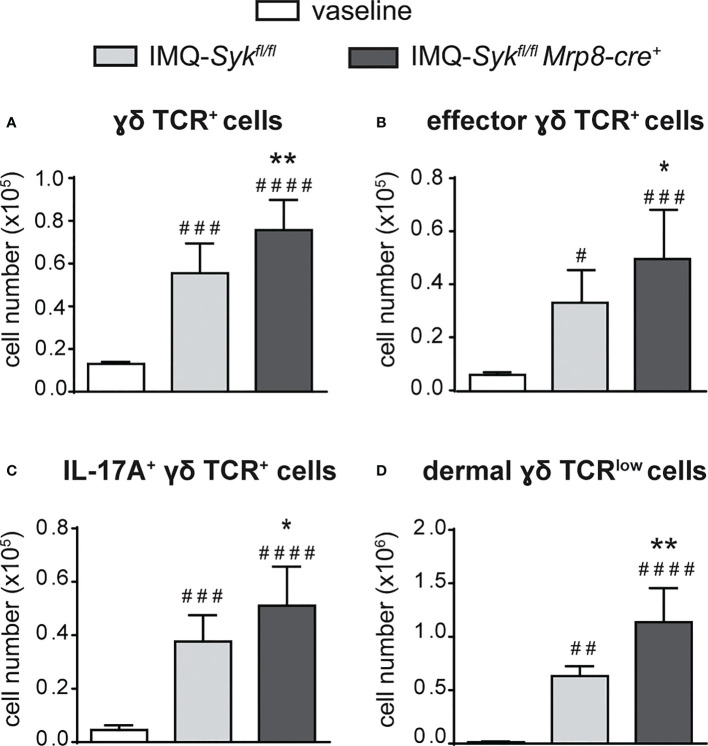
Infiltration of neutrophils and γδ T cells in the draining lymph nodes and skin of IMQ-treated *Syk^fl/fl^* and *Syk^fl/fl^Mrp8-cre^+^* mice. The dorsal skin of control *Syk^fl/fl^* and *Syk^fl/fl^Mrp8-cre^+^* mice was topically treated with IMQ-containing cream (Aldara^®^) or vaseline for 6 consecutive days. Draining lymph nodes **(A-C)** or the total skin (2x2 cm) **(D)** were collected and analyzed by flow cytometry. Panels report: the number of total (**A**), effector (CD44^high^ CD62L^low^) **(B)** and IL-17A-producing **(C)** lymph node γδ TCR^+^ cells; the number of skin dermal γδ TCR^low^ T cells (**D**). Data are pooled from 2 separate experiments and are expressed as means ± SD (n = 11 mice). Statistical differences of IMQ-treated *Syk^fl/fl^* or *Syk^fl/fl^Mrp8-cre^+^vs*. vaseline-treated mice (#) and IMQ-treated *Syk^fl/fl^vs*. IMQ-treated *Syk^fl/fl^Mrp8-cre^+^* mice (*) are reported. *P ≤ 0.05; **P ≤ 0.01; #*P* ≤ 0.05; ##*P* ≤ 0.01; ###*P* ≤ 0.001; #### *P* ≤ 0.0001 by 1-way ANOVA with Bonferroni’s post-test.

We next performed the IMQ-induced psoriasis model in *Syk^fl/fl^MRP8-cre^+^* mice, to evaluate the effect of Syk-deficiency in neutrophils on disease development. Histological section measurement of dorsal skin in *Syk^fl/fl^MRP8-cre^+^* mice failed to show significant variations in epidermal thickness as compared to control mice (consisting of a mix of *Syk^+/+^MRP8-cre^+^Syk^+/+^MRP8-cre^-^* mice) after 6 days of IMQ-treatment (**Figure 8A**). However, similarly to neutrophil-depleted mice, *Syk^fl/fl^ MRP8-cre^+^* mice manifested an enhanced expression of skin-associated psoriatic genes, such as S100A7 and Lcn2, as well as a specific increase in the expression of cytokines implicated in the IL-23/T17 axis, including IL-23, IL-22, IL-17, CXCL1 and IL-6 after 6 days of IMQ treatment (**Figure 8B** and data not shown). Furthermore, also the number of total and activated γδ T cells producing IL-17 (**Figures 9A–C**), was increased in the draining lymph nodes of *Syk^fl/fl^ MRP8-cre^+^* mice, as compared to control mice, after 6 days of IMQ treatment. In a similar fashion, the number of dermal γδ T cells (**Figure 9D**) infiltrating into the skin of IMQ-treated of *Syk^fl/fl^ MRP8-cre^+^* mice was significantly increased as compared to IMQ-treated control mice.

It is noteworthy to remark that, in line with the fact that Syk is not directly involved in controlling neutrophil migration to the inflammatory sites (51), we did not notice any significant difference in the capacity of *Syk^-/-^* neutrophils to infiltrate the lymph nodes and the skin in response to IMQ treatment (**Supplementary Figures 4A, B**).

Overall, data suggest that Syk-dependent signaling pathways controlling neutrophil effector functions, but not neutrophil migration, are required for neutrophil-mediated inhibition of γδ T functions *in vitro*, and in IMQ-induced psoriasis *in vivo*.

## Discussion

Neutrophil accumulation in the skin is one of the histological features that characterize psoriasis (1, 2). However, the role of neutrophils in psoriasis development remains poorly understood. In this study, by utilizing the mouse model of IMQ-induced psoriasis, we uncover a novel role of neutrophils as negative regulators of disease propagation and exacerbation. In fact, neutrophil depletion resulted in an increased epidermal thickening accompanied by an increased inflammatory cell infiltration and cytokine/psoriatic gene overexpression. In particular, neutrophil depletion resulted in a profound exacerbation of the inflammation associated with the IL-23/T17 pathway. Interestingly, such an effect seemed to be mediated by the ability of neutrophils to inhibit *via* contact- and NADPH oxidase-dependent ROS production, the proliferation and the production of IL-17 by γδ T cells. Finally, we demonstrated for the first time that Syk-based signaling in neutrophils plays a crucial role in the inhibitory crosstalk between neutrophils and γδ T cells. The relevance of the latter finding is supported by the fact that, like neutrophil-depleted mice, mice carrying a selective impairment of Syk-dependent signaling only in neutrophils manifested an exacerbated skin inflammation and γδ T cell infiltration in response to IMQ treatment.

Neutrophils, due to their ability to both promote and inhibit inflammatory and immune responses, seem to play a rather complex role in several inflammatory diseases (14, 16–18, 52, 53). As far as psoriasis, the current hypothesis is that neutrophils play a pro-inflammatory role in disease pathogenesis (13, 20). This assumption is mostly based on the fact that these cells are generally linked to the IL-23/T17-related inflammatory axis and that they have been proposed to sustain skin inflammation, for example, by producing NETs (54, 55) and other inflammatory cytokines [(including IL-17 and IL-22 (56)] or by activating IL-36 family cytokines *via* the release of proteases (57). However, to date, compelling evidence of this pathogenic role of neutrophils in psoriasis does not exist. For instance, neutrophils have been proposed to play a regulatory role in psoriatic inflammation *via* the release of elastase and the consequent activation of the anti-inflammatory cytokine IL-36 receptor antagonist (58), a negative modulator of psoriasis development (58). Few studies have attempted to clarify the pathogenic role of neutrophils in disease pathogenesis by utilizing different types of preclinical model (19, 24–26). In flaky skin mice (*fsn/fsn*), which spontaneously develop psoriasis-like disease, neutrophils were proposed to be pro-inflammatory and to promote psoriasis development (19). However, it is difficult to draw definitive conclusions on the specific role played by neutrophils in this model, given that the study was performed by utilizing depleting or blocking Abs not specific for neutrophils [e.g. anti-GR1 Ab (clone RB6-8C5) or anti-α_M_β_2_ (CD11b/CD18; clone M1/70) Ab] (19). Similar to our work, three additional studies have instead attempted to perform neutrophil depletion by utilizing the specific anti-Ly6G Ab (clone 1A8) in the IMQ-induced mouse model of psoriasis (24–26). However, contradictory findings were reported, since in the studies by Sumida H. et al. (25) and Han G. et al. (26) neutrophils were shown to be proinflammatory and to contribute to psoriasis development, in the study by Singh T. et al. (24) neutrophils were shown not to affect disease development, while we found a protective role for neutrophils. The reasons for these controversial results can be likely attributed to the fact that several variations to the original protocol for IMQ-induced psoriasis (e.g. Aldara dosage, treatment of back skin *versus* ears, total day of treatment, mouse strain utilized, etc.) have been utilized across different laboratories (27). For instance, we choose to perform the mostly utilized protocol originally published by van der Fits, L. et al. (application of 60 mg of Aldara cream on the shaved back for 6 days (28), in the study performed by Sumida, H. et al. the induction of the disease involved the application of a lower dose of Aldara cream (30 mg) on the shaved back for 6 days (25), whereas in the study by Singh, T. et al. the authors applied even a lower dose of Aldara cream (25 mg) on mice, for a shorter period of time (4 days), and on the ears instead of the shaved back (24). In the study performed by Han G et al, although the protocol utilized was the same as the one utilized in our study, BALB/c instead of CD57BL/6 mice were utilized (26). The different housing conditions of the animal facilities may have also influenced the controversial results among the three studies. However, we tend to exclude this possibility as neutrophil-depleted mice treated with our experimental protocol of IMQ-induced psoriasis and housed in a different animal facility (University of California, San Francisco, USA) displayed a similar enhancement of epidermal thickening after 6 days of IMQ treatment (C.A. and C.A.L. unpublished observation).

Despite these limitations, likely intrinsic to the peculiar experimental model and conditions utilized, the important message emerging from our study is that neutrophils may acquire a regulatory role during psoriasis development throughout their preferential interactions with γδ T cells. Our data suggest indeed that, at least in this IMQ-mouse model of psoriasis, the capability of neutrophils to inhibit γδ T cell functions at late disease-stages is more relevant to disease progression than the intrinsic capability of these cells to contribute to skin inflammation *via* the production of cytokines and other inflammatory mediators. In this context, controversial observations on the crosstalk occurring between neutrophils and γδ T cells were reported in the last decade (16, 59). For example, human neutrophils were shown to either stimulate γδ T cells (60) or negatively modulate γδ T-cell activation (41, 42). Also in mice, pieces of evidence that neutrophils can both inhibit (44, 61), or stimulate the proliferation and IL-17 production by γδ T cells do exist (62). The interactions between neutrophils and γδ T cells have been proposed to be mediated *via* the release of serine proteases (41, 43) or the production of ROS (42, 44). Our findings demonstrate that the inhibitory effect of murine neutrophils on γδ T cells is dependent on cell contacts and mediated by NADPH oxidase activation and ROS release, in agreement with two other reports by Sabbione et al. (42) and Mensurado et al. (44) that used human and mouse neutrophils, respectively. In addition, we propose, for the first time, Syk as important signaling molecule involved in the modulation of this inhibitory pathway. Considering the important role of Syk in mediating integrin-dependent functions (51), and that the capability of other populations of suppressive neutrophils to inhibit the proliferation and interferon γ (IFNγ) production by T cells *via* a CD18-mediated contact-dependent mechanism has been previously described (47, 48), our data suggest that syk-dependent signaling may be involved in the modulation of this integrin-mediated neutrophil inhibitory function in the inflammatory microenvironment. Future studies should further address this issue.

γδ T cells are known to be particularly susceptible to oxidative stress (63). Interestingly, several evidences support the contribution of IL-17 in Chronic Granulomatous Disease (CGD) mediated hyperinflammation (64) and susceptibility to autoimmune diseases (65). Even though these phenomena have been so far mostly linked to an expansion of Th7 lymphocytes (66), the possible contribution of γδ T cells is also starting to emerge. In this context, a strong response of IL-17–producing γδ T cells was reported in *p47^phox-/-^* mice infected with *A. fumigatus* (67). Most relevant to the current study is the finding that *p47^phox-/-^* mice were reported to develop enhanced IMQ-induced psoriasis (68). However, future experimental evidence will be fundamental to further characterize the specific role of neutrophil-mediated inhibition of γδ T cell functions in this phenomenon. Overall, our study proposes that neutrophils can act as important negative regulators in the IMQ-mediated model of psoriasis, instead of promoting inflammation. Considering that psoriasis consists of a heterogeneous type of disease where each of its individual clinical phenotypes represents a different balance between autoimmune and autoinflammatory immune processes, it may be worth verifying the effective role of neutrophils also in human psoriasis. Neutrophils may indeed act as unexpected negative players of disease development in specific types or clinical stages of human psoriasis. A better understanding of the specific role of human neutrophils in psoriasis is mostly hampered by fact that this disease is often associated to a various range of co-morbidities (69) that may affect the phenotype of circulating neutrophils. However, to gain more mechanistic insights into the role of neutrophils in human psoriasis would be extremely important to facilitate the design of novel therapeutic strategies for the clinical management of this pathological condition. Similarly to what was reported in the preclinical models of psoriasis, also the pivotal role of IL-17-producing γδ T cells in human psoriasis started to emerge (31, 70), indicating that possible crosstalk between neutrophils and γδ T cells may exist also in humans, and may play a potential role in the modulation of disease development.

## Data availability statement

The raw data supporting the conclusions of this article will be made available by the authors, without undue reservation.

## Ethics statement

The animal study was reviewed and approved by the Ethics Committee for the usage of laboratory animals for research purposes at the University of Verona and by the Italian Ministry of Health (approval 339/2015-PR).

## Author contributions

SC, DB, and PS designed the research study and performed data analysis. SC, DB, EC, OM, SG, FP, MD, SL, CLA, PR, FDS, and TC performed experiments. GG, FT, WV, EZ, SU, GC, and SD provided intellectual guidance. CL, MC, and PS wrote the paper. All authors contributed to the article and approved the submitted version.

## Funding

This work was supported by grants from: Università di Verona (RBVR17NCNC to PS); Associazione Italiana per la Ricerca sul Cancro (AIRC, IG20339 to MC and AIRC IG-23179 to WV); Ministero dell’Istruzione, dell’Università e della Ricerca (PRIN 2015YYKPNN to MC); European Research Council (ERC) advanced grant no. 695714 IMMUNOALZHEIMER and the ERC Proof of Concept grant nr. 101069397 NeutrAD (to GC); European Cooperation in Science and Technology (COST) Actions BM1404 Mye-EUNITER (www.mye-euniter.eu). COST is supported by the EU Framework Program Horizon 2020.

## Acknowledgments

We thank S. Zini (supported by Fondazione Beretta, University of Brescia) for her important contribution to the immunohistochemical staining experiments. We thank Prof. L. Romani (University of Perugia) for providing *p47^phox-/-^* mice.

## Conflict of interest

The authors declare that the research was conducted in the absence of any commercial or financial relationships that could be construed as a potential conflict of interest.

## Publisher’s note

All claims expressed in this article are solely those of the authors and do not necessarily represent those of their affiliated organizations, or those of the publisher, the editors and the reviewers. Any product that may be evaluated in this article, or claim that may be made by its manufacturer, is not guaranteed or endorsed by the publisher.

## Abbreviations

(DCs), Dendritic cells; (T17), IL-17- producing T cell subsets;(IL-23), interleukin 23; (NADPH), nicotinamide adenine dinucleotide phosphate; (ROS), reactive oxygen species; (Th17), T helper 17; (WT), wild-type; (Syk), Spleen tyrosine kinase; (FcgRs), Fcg receptors; (DPI), diphenyleneiodonium; (PTX), including pentoxyfilline; (ARG1), arginase-1; (PSGL-1), P-Selectin glycoprotein ligand 1.

## References

[1] Boehncke WH Schon MP. Psoriasis. Lancet (2015) 386(9997):983–94. doi: 10.1016/S0140-6736(14)61909-7 26025581

[2] Deng Y Chang C Lu Q. The inflammatory response in psoriasis: a comprehensive review. Clin Rev Allergy Immunol (2016) 50:377–89. doi: 10.1007/s12016-016-8535-x 27025861

[3] Hawkes JE Gudjonsson JE Ward NL. The snowballing literature on imiquimod-induced skin inflammation in mice: A critical appraisal. J Invest Dermatol (2016). doi: 10.1016/j.jid.2016.10.024 PMC5326590 27955901

[4] Vicic M Kastelan M Brajac I Sotosek V Massari LP. Current concepts of psoriasis immunopathogenesis. Int J Mol Sci (2021) 22(21):11574–88. doi: 10.3390/ijms222111574 PMC8584028 34769005

[5] Bugaut H Aractingi S. Major role of the IL17/23 axis in psoriasis supports the development of new targeted therapies. Front Immunol (2021) 12:621956. doi: 10.3389/fimmu.2021.621956 33717124 PMC7948519

[6] Mease PJ. Inhibition of interleukin-17, interleukin-23 and the TH17 cell pathway in the treatment of psoriatic arthritis and psoriasis. Curr Opin Rheumatol (2015) 27(2):127–33. doi: 10.1097/BOR.0000000000000147 25599143

[7] Castillo-Gonzalez R Cibrian D Sanchez-Madrid F. Dissecting the complexity of gammadelta T-cell subsets in skin homeostasis, inflammation, and malignancy. J Allergy Clin Immunol (2021) 147(6):2030–42. doi: 10.1016/j.jaci.2020.11.023 33259837

[8] Diani M Altomare G Reali E. T Cell responses in psoriasis and psoriatic arthritis. Autoimmun Rev (2015) 14(4):286–92. doi: 10.1016/j.autrev.2014.11.012 25445403

[9] Hu P Wang M Gao H Zheng A Li J Mu D. The role of helper T cells in psoriasis. Front Immunol (2021) 12:788940. doi: 10.3389/fimmu.2021.788940 34975883 PMC8714744

[10] Jariwala SP. The role of dendritic cells in the immunopathogenesis of psoriasis. Arch Dermatol Res (2007) 299(8):359–66. doi: 10.1007/s00403-007-0775-4 PMC1978540 17680257

[11] Wang A Bai Y. Dendritic cells: The driver of psoriasis. J Dermatol (2020) 47(2):104–13. doi: 10.1111/1346-8138.15184 31833093

[12] Coimbra S Figueiredo A Castro E Rocha-Pereira P Santos-Silva A. The roles of cells and cytokines in the pathogenesis of psoriasis. Int J Dermatol (2012) 51(4):389–95. doi: 10.1111/j.1365-4632.2011.05154.x 22435425

[13] Schon MP Broekaert SM Erpenbeck L. Sexy again: The renaissance of neutrophils in psoriasis. Exp Dermatol (2017) 26(4):305–11. doi: 10.1111/exd.13067 27194625

[14] Nicolas-Avila JA Adrover JM Hidalgo A. Neutrophils in homeostasis, immunity, and cancer. Immunity (2017) 46(1):15–28. doi: 10.1016/j.immuni.2016.12.012 28099862

[15] Mantovani A Cassatella MA Costantini C Jaillon S. Neutrophils in the activation and regulation of innate and adaptive immunity. Nat Rev Immunol (2011) 11(8):519–31. doi: 10.1038/nri3024 21785456

[16] Scapini P Cassatella MA. Social networking of human neutrophils within the immune system. Blood (2014) 124(5):710–9. doi: 10.1182/blood-2014-03-453217 24923297

[17] Scapini P Marini O Tecchio C Cassatella MA. Human neutrophils in the saga of cellular heterogeneity: Insights and open questions. Immunol Rev (2016) 273(1):48–60. doi: 10.1111/imr.12448 27558327

[18] Soehnlein O Steffens S Hidalgo A Weber C. Neutrophils as protagonists and targets in chronic inflammation. Nat Rev Immunol (2017) 17(4):248–61. doi: 10.1038/nri.2017.10 28287106

[19] Schon M Denzer D Kubitza RC Ruzicka T Schon MP. Critical role of neutrophils for the generation of psoriasiform skin lesions in flaky skin mice. J Invest Dermatol (2000) 114(5):976–83. doi: 10.1046/j.1523-1747.2000.00953.x 10771480

[20] Chiang CC Cheng WJ Korinek M Lin CY Hwang TL. Neutrophils in psoriasis. Front Immunol (2019) 10:2376. doi: 10.3389/fimmu.2019.02376 31649677 PMC6794444

[21] Pai S Balasubramanian R Shenoi S Sandra A. Clearance of psoriasis following agranulocytosis. Int J Dermatol (1999) 38(11):876–70. 10583941

[22] Toichi E Tachibana T Furukawa F. Rapid improvement of psoriasis vulgaris during drug-induced agranulocytosis. J Am Acad Dermatol (2000) 43(2 Pt 2):391–5. doi: 10.1067/mjd.2000.103264 10901732

[23] Bhushan M Bleiker TO Ballsdon AE Allen MH Sopwith M Robinson MK. Anti-e-selectin is ineffective in the treatment of psoriasis: a randomized trial. Br J Dermatol (2002) 146(5):824–31. doi: 10.1046/j.1365-2133.2002.04743.x 12000379

[24] Singh TP Zhang HH Borek I Wolf P Hedrick MN Singh SP. Monocyte-derived inflammatory langerhans cells and dermal dendritic cells mediate psoriasis-like inflammation. Nat Commun (2016) 7:13581. doi: 10.1038/ncomms13581 27982014 PMC5171657

[25] Sumida H Yanagida K Kita Y Abe J Matsushima K Nakamura M. Interplay between CXCR2 and BLT1 facilitates neutrophil infiltration and resultant keratinocyte activation in a murine model of imiquimod-induced psoriasis. J Immunol (2014) 192(9):4361–9. doi: 10.4049/jimmunol.1302959 24663678

[26] Han G Havnaer A Lee HH Carmichael DJ Martinez LR. Biological depletion of neutrophils attenuates pro-inflammatory markers and the development of the psoriatic phenotype in a murine model of psoriasis. Clin Immunol (2020) 210:108294. doi: 10.1016/j.clim.2019.108294 31678366 PMC8043213

[27] Flutter B Nestle FO. TLRs to cytokines: mechanistic insights from the imiquimod mouse model of psoriasis. Eur J Immunol (2013) 43(12):3138–46. doi: 10.1002/eji.201343801 24254490

[28] van der Fits L Mourits S Voerman JS Kant M Boon L Laman JD. Imiquimod-induced psoriasis-like skin inflammation in mice is mediated *via* the IL-23/IL-17 axis. J Immunol (2009) 182(9):5836–45. doi: 10.4049/jimmunol.0802999 19380832

[29] Garzorz-Stark N Lauffer F Krause L Thomas J Atenhan A Franz R. Toll-like receptor 7/8 agonists stimulate plasmacytoid dendritic cells to initiate TH17-deviated acute contact dermatitis in human subjects. J Allergy Clin Immunol (2017) 141:1320–33. doi: 10.1016/j.jaci.2017.07.045 28935206

[30] Pantelyushin S Haak S Ingold B Kulig P Heppner FL Navarini AA. Rorgammat+ innate lymphocytes and gammadelta T cells initiate psoriasiform plaque formation in mice. J Clin Invest (2012) 122(6):2252–6. doi: 10.1172/JCI61862 PMC3366412 22546855

[31] Cai Y Shen X Ding C Qi C Li K Li X. Pivotal role of dermal IL-17-producing gammadelta T cells in skin inflammation. Immunity (2011) 35(4):596–610. doi: 10.1016/j.immuni.2011.08.001 21982596 PMC3205267

[32] Wohn C Ober-Blobaum JL Haak S Pantelyushin S Cheong C Zahner SP. Langerin(neg) conventional dendritic cells produce IL-23 to drive psoriatic plaque formation in mice. Proc Natl Acad Sci U.S.A. (2013) 110(26):10723–8. doi: 10.1073/pnas.1307569110 PMC3696803 23754427

[33] Yoshiki R Kabashima K Honda T Nakamizo S Sawada Y Sugita K. IL-23 from langerhans cells is required for the development of imiquimod-induced psoriasis-like dermatitis by induction of IL-17A-producing gammadelta T cells. J Invest Dermatol (2014) 134(7):1912–21. doi: 10.1038/jid.2014.98 24569709

[34] Tortola L Rosenwald E Abel B Blumberg H Schafer M Coyle AJ. Psoriasiform dermatitis is driven by IL-36-mediated DC-keratinocyte crosstalk. J Clin Invest (2012) 122(11):3965–76. doi: 10.1172/jci63451 PMC3484446 23064362

[35] Jackson SH Gallin JI Holland SM. The p47phox mouse knock-out model of chronic granulomatous disease. J Exp Med (1995) 182(3):751–8. doi: 10.1084/jem.182.3.751 PMC2192153 7650482

[36] Elliott ER Van Ziffle JA Scapini P Sullivan BM Locksley RM Lowell CA. Deletion of syk in neutrophils prevents immune complex arthritis. J Immunol (2011) 187(8):4319–30. doi: 10.4049/jimmunol.1100341 PMC3186826 21918195

[37] Van Ziffle JA Lowell CA. Neutrophil-specific deletion of syk kinase results in reduced host defense to bacterial infection. Blood (2009) 114(23):4871–82. doi: 10.1182/blood-2009-05-220806 PMC2786293 19797524

[38] Costa S Marini O Bevilacqua D DeFranco AL Hou B Lonardi S. Role of MyD88 signaling in the imiquimod-induced mouse model of psoriasis: focus on innate myeloid cells. J Leukoc Biol (2017) 102(3):791–803. doi: 10.1189/jlb.3MA0217-054RR 28642279 PMC6608051

[39] Scapini P Lamagna C Hu Y Lee K Tang Q DeFranco AL. B cell-derived IL-10 suppresses inflammatory disease in Lyn-deficient mice. Proc Natl Acad Sci U.S.A. (2011) 108(41):E823–32. doi: 10.1073/pnas.1107913108 PMC3193193 21911371

[40] Tamassia N Cassatella MA Bazzoni F. Fast and accurate quantitative analysis of cytokine gene expression in human neutrophils by reverse transcription real-time PCR. Methods Mol Biol (2020) 2087:243–60. doi: 10.1007/978-1-0716-0154-9_19 31728997

[41] Fazio J Kalyan S Wesch D Kabelitz D. Inhibition of human gammadelta T cell proliferation and effector functions by neutrophil serine proteases. Scand J Immunol (2014) 80(6):381–9. doi: 10.1111/sji.12221 25345993

[42] Sabbione F Gabelloni ML Ernst G Gori MS Salamone G Oleastro M. Neutrophils suppress gammadelta T-cell function. Eur J Immunol (2014) 44(3):819–30. doi: 10.1002/eji.201343664 24271816

[43] Towstyka NY Shiromizu CM Keitelman I Sabbione F Salamone GV Geffner JR. Modulation of gammadelta T-cell activation by neutrophil elastase. Immunology (2017) 153:225–37. doi: 10.1111/imm.12835 PMC5765375 28888033

[44] Mensurado S Rei M Lanca T Ioannou M Goncalves-Sousa N Kubo H. Tumor-associated neutrophils suppress pro-tumoral IL-17+ gammadelta T cells through induction of oxidative stress. PloS Biol (2018) 16(5):e2004990. doi: 10.1371/journal.pbio.2004990 29750788 PMC5965901

[45] Schmielau J Finn OJ. Activated granulocytes and granulocyte-derived hydrogen peroxide are the underlying mechanism of suppression of t-cell function in advanced cancer patients. Cancer Res (2001) 61(12):4756–60. 11406548

[46] Choi J Suh B Ahn YO Kim TM Lee JO Lee SH. CD15+/CD16low human granulocytes from terminal cancer patients: Granulocytic myeloid-derived suppressor cells that have suppressive function. Tumour Biol (2012) 33(1):121–9. doi: 10.1007/s13277-011-0254-6 22081309

[47] Pillay J Kamp VM van Hoffen E Visser T Tak T Lammers JW. A subset of neutrophils in human systemic inflammation inhibits T cell responses through mac-1. J Clin Invest (2012) 122(1):327–36. doi: 10.1172/JCI57990 PMC3248287 22156198

[48] Marini O Costa S Bevilacqua D Calzetti F Tamassia N Spina C. Mature CD10(+) and immature CD10(-) neutrophils present in G-CSF-treated donors display opposite effects on T cells. Blood (2017) 129(10):1343–56. doi: 10.1182/blood-2016-04-713206 28053192

[49] Stadtmann A Germena G Block H Boras M Rossaint J Sundd P. The PSGL-1-L-selectin signaling complex regulates neutrophil adhesion under flow. J Exp Med (2013) 210(11):2171–80. doi: 10.1084/jem.20130664 PMC3804951 24127491

[50] Futosi K Fodor S Mocsai A. Neutrophil cell surface receptors and their intracellular signal transduction pathways. Int Immunopharmacol (2013) 17(3):638–50. doi: 10.1016/j.intimp.2013.06.034 PMC3827506 23994464

[51] Mocsai A Zhou M Meng F Tybulewicz VL Lowell CA. Syk is required for integrin signaling in neutrophils. Immunity (2002) 16(4):547–58. doi: 10.1016/S1074-7613(02)00303-5 11970878

[52] Lehman HK Segal BH. The role of neutrophils in host defense and disease. J Allergy Clin Immunol (2020) 145(6):1535–44. doi: 10.1016/j.jaci.2020.02.038 PMC8912989 32283205

[53] Liew PX Kubes P. The neutrophil’s role during health and disease. Physiol Rev (2019) 99(2):1223–48. doi: 10.1152/physrev.00012.2018 30758246

[54] Zabieglo K Majewski P Majchrzak-Gorecka M Wlodarczyk A Grygier B Zegar A. The inhibitory effect of secretory leukocyte protease inhibitor (SLPI) on formation of neutrophil extracellular traps. J Leukoc Biol (2015) 98(1):99–106. doi: 10.1189/jlb.4AB1114-543R 25917460 PMC4467168

[55] Lin AM Rubin CJ Khandpur R Wang JY Riblett M Yalavarthi S. Mast cells and neutrophils release IL-17 through extracellular trap formation in psoriasis. J Immunol (2011) 187(1):490–500. doi: 10.4049/jimmunol.1100123 21606249 PMC3119764

[56] Dyring-Andersen B Honore TV Madelung A Bzorek M Simonsen S Clemmensen SN. Skov l. interleukin (IL)-17A and IL-22-producing neutrophils in psoriatic skin. Br J Dermatol (2017) 177:e321–2. doi: 10.1111/bjd.15533 PMC5921865 28369663

[57] Henry CM Sullivan GP Clancy DM Afonina IS Kulms D Martin SJ. Neutrophil-derived proteases escalate inflammation through activation of IL-36 family cytokines. Cell Rep (2016) 14(4):708–22. doi: 10.1016/j.celrep.2015.12.072 26776523

[58] Macleod T Doble R McGonagle D Wasson CW Alase A Stacey M. Neutrophil elastase-mediated proteolysis activates the anti-inflammatory cytokine IL-36 receptor antagonist. Sci Rep (2016) 6:24880. doi: 10.1038/srep24880 27101808 PMC4840362

[59] Kalyan S Chandrasekaran V Quabius ES Lindhorst TK Kabelitz D. Neutrophil uptake of nitrogen-bisphosphonates leads to the suppression of human peripheral blood gammadelta T cells. Cell Mol Life Sci (2014) 71(12):2335–46. doi: 10.1007/s00018-013-1495-x PMC11114071 24162933

[60] Towstyka NY Shiromizu CM Keitelman I Sabbione F Salamone GV Geffner JR. Modulation of gammadelta T-cell activation by neutrophil elastase. Immunology (2018) 153(2):225–37. doi: 10.1111/imm.12835 PMC5765375 28888033

[61] Wozniak KL Kolls JK Wormley FL Jr. Depletion of neutrophils in a protective model of pulmonary cryptococcosis results in increased IL-17A production by gammadelta T cells. BMC Immunol (2012) 13:65. doi: 10.1186/1471-2172-13-65 23216912 PMC3538069

[62] Hassane M Demon D Soulard D Fontaine J Keller LE Paget C. Neutrophilic NLRP3 inflammasome-dependent IL-1beta secretion regulates the gammadeltaT17 cell response in respiratory bacterial infections. Mucosal Immunol (2017) 10(4):1056–68. doi: 10.1038/mi.2016.113 28051086

[63] Marlin R Pappalardo A Kaminski H Willcox CR Pitard V Netzer S. Sensing of cell stress by human gammadelta TCR-dependent recognition of annexin A2. Proc Natl Acad Sci U.S.A. (2017) 114(12):3163–8. doi: 10.1073/pnas.1621052114 PMC5373368 28270598

[64] Rieber N Hector A Kuijpers T Roos D Hartl D. Current concepts of hyperinflammation in chronic granulomatous disease. Clin Dev Immunol (2012) 2012:252460. doi: 10.1155/2012/252460 21808651 PMC3144705

[65] De Ravin SS Naumann N Cowen EW Friend J Hilligoss D Marquesen M. Chronic granulomatous disease as a risk factor for autoimmune disease. J Allergy Clin Immunol (2008) 122(6):1097–103. doi: 10.1016/j.jaci.2008.07.050 PMC2786235 18823651

[66] Horvath R Rozkova D Lastovicka J Polouckova A Sedlacek P Sediva A. Expansion of T helper type 17 lymphocytes in patients with chronic granulomatous disease. Clin Exp Immunol (2011) 166(1):26–33. doi: 10.1111/j.1365-2249.2011.04449.x 21910722 PMC3193916

[67] Romani L Fallarino F De Luca A Montagnoli C D’Angelo C Zelante T. Defective tryptophan catabolism underlies inflammation in mouse chronic granulomatous disease. Nature (2008) 451(7175):211–5. doi: 10.1038/nature06471 18185592

[68] Kim HR Lee A Choi EJ Hong MP Kie JH Lim W. Reactive oxygen species prevent imiquimod-induced psoriatic dermatitis through enhancing regulatory T cell function. PloS One (2014) 9(3):e91146. doi: 10.1371/journal.pone.0091146 24608112 PMC3946742

[69] Korman NJ. Management of psoriasis as a systemic disease: what is the evidence? Br J Dermatol (2020) 182(4):840–8. doi: 10.1111/bjd.18245 PMC7187293 31225638

[70] Laggner U Di Meglio P Perera GK Hundhausen C Lacy KE Ali N. Identification of a novel proinflammatory human skin-homing Vgamma9Vdelta2 T cell subset with a potential role in psoriasis. J Immunol (2011) 187(5):2783–93. doi: 10.4049/jimmunol.1100804 PMC3187621 21813772

