# Supplementary material for: Neutrophils inhibit γδ T cell functions in the imiquimod-induced mouse model of psoriasis
Source: Front Immunol. 2022 Nov 15;13:1049079. doi: 10.3389/fimmu.2022.1049079 (PMC9709482; doi:10.3389/fimmu.2022.1049079)
Supplement: Supplementary file 1 [file DataSheet_1.docx]

**SUPPLEMENTARY MATERIALS for**

**Neutrophils inhibit γδ T cell functions in the imiquimod-induced mouse model of psoriasis**

Sara Costa^†1^, Dalila Bevilacqua^†1^, Elena Caveggion^1^, Sara Gasperini^1^, Elena Zenaro^1^, Francesca Pettinella^1^, Marta Donini^1^, Stefano Dusi^1^, Gabriela Constantin^1^, Silvia Lonardi^4^, William Vermi^4^, Francesco De Sanctis^2^, Stefano Ugel^2^, Tiziana Cestari ^2^, Clare L. Abram^5^, Clifford A. Lowell^5^, Pamela Rodegher^6|^, Franco Tagliaro^6^, Giampiero Girolomoni^3^, Marco A. Cassatella^1^ and Patrizia Scapini^1*^

^†^ these authors contributed equally to this work and share first authorship

^*^Correspondence should be addressed to Patrizia Scapini: patrizia.scapini@univr.it

**SUPPLEMENTARY METHODS**

**Isolation of peritoneal neutrophils**

Peritoneal exudates were recovered 16 hours after i.p. injection of Bio-Gel® P Polyacrylamide Beads (Bio Rad). After a passage through a 70 μM cell strainer, cell suspensions were incubated with different mAbs as described in the flow cytometry section. CD11b^+^Ly6C^low^Ly6G^high^ neutrophils were sorted using a FACS AriaTM II flow cytometer (Becton Dickinson) (> 99.0 % purity). Alternatively, neutrophils were purified using EasySep™ Mouse Neutrophil Enrichment Kit according to the manifacturer’s instructions (>95% purity). Purified cells were suspended at proper concentration depending on the assay in RPMI 1640 medium supplemented with 10 % FBS, 1 % ultraglutammine and 1 % penicillin/streptomycin (BioWhittaker-Lonza), added to 96-well plates and cultured at 37˚C, 5 % CO_2_.

**Proliferation and IL-17A production by γδ T cells**

γδ T cells were isolated from single-cell suspensions of mouse spleen and lymph nodes from wild-type or *Tcra^-/-^* mice, using the TCRγ/δ^+^ T Cell Isolation Kit (Miltenyi Biotec) (> 85.5 % purity). Proliferation assay was performed in 96-well plates pre-coated (for 1 hour at 37˚C) with 1 µg ml^-1^ anti-CD3 mAbs [(G23-8, eBioscience). 1 x 10^5^ γδ T cells /well and 2 µg/ml anti-CD28 mAbs (B122, eBioscience) were then added to the plates (at 37˚C, 5 % CO_2_), in the presence of 10 ng/mL IL-1β (eBioscience) *plus* 100 ng/mL IL-23 (eBioscience). After 24 hours purified peritoneal neutrophils were added to the culture at the appropriate ratio. Following a 72 hour-incubation BrdU was added to the co-cultures for additional 4 hours; supernatants were then harvested for measurement of IL- 17A by using a specific ELISA kit (R&D Systems) while proliferation was determined by BrDU incorporation by ELISA (Cell proliferation ELISA, Roche), following the manufacturer’s protocol. γδ T cell inhibition of proliferation is expressed as percentage of decrease of the absorbance value (at 450 nm: revealing the amount of BrdU incorporated in proliferating γδ T cells) of each experimental conditions over the absorbance value of anti-CD3/CD28-stimulated γδ T cells.

**Quantification of reactive oxygen species**

Oxidative stress detection was performed using the cellROX® Deep Red Flow Cytometry Assay Kit (Life Technology). 96-well plates were pre-coated (for 1 hour at 37˚C) with 1 µg ml^-1^ anti-CD3 mAbs [(G23-8, eBioscience). 1 x 10^5^ γδ T cells/well and 2 µg/ml anti-CD28 mAbs (B122, eBioscience) were added into the plates (at 37˚C, 5 % CO_2_), in the presence of 10 ng/mL IL-1β (eBioscience) *plus* 100 ng/mL IL-23 (eBioscience). The day after purified peritoneal neutrophils were added to the culture at 1:1 γδ T cells-to-neutrophils ratio for 1,5 hours. Then cells were stained with the CellROX^®^ Deep Red reagent for additional 1.5 hours. Sample fluorescence was measured by a seven-colour MACSQuant Analyzer (Miltenyi Biotec), while data analysis was performed by using FlowJo software Version 8.8.6 (Tree Star, Ashland, OR, USA).

**Supplementary Table 1:** **List of the gene-specific primer pairs utilized**

| **Gene Name** | **Forward Primers** | **Reverse Primers** |
| --- | --- | --- |
| **IL-36a** | TGCCCACTCATTCTGACCCA | GTGCCACAGAGCAATGTGTC |
| **IL-1α** | CAGTGAAATTTGACATGGGTG | CAGGCATCTCCTTCAGCAG |
| **S100A7** | GCCTCGCTTCATGGACAC | CGGAACAGCTCTGTGATGTAGT |
| **Lcn2** | GGACCAGGGCTGTCGCTACT | GGTGGCCACTTGCACATTGT |
| **IL-17 A** | ATCAGGACGCGCAAACATGA | TTGGACACGCTGAGCTTTGA |
| **IL-22** | TGACGACCAGAACATCCAGA | CGCCTTGATCTCTCCACTCT |
| **IL‐6** | CCACTTCACAAGTCGGAGGCTTA | GCAAGTGCATCATCGTTGTTCATAC |
| **IL-23p19** | CACCTCCCTACTAGGACTCAGC | CTGCCACTGCTGACTAGAAC |
| **IL-1β** | AGATGAAGTGCTCCTTCCAG | TTGTCCATGGCCACAACAAC |
| **CXCL-1** | ACCCAAACCGAAGTCATAGC | TTTCTCCGTTACTTGGGGAC |

**SUPPLEMENTARY FIGURES**


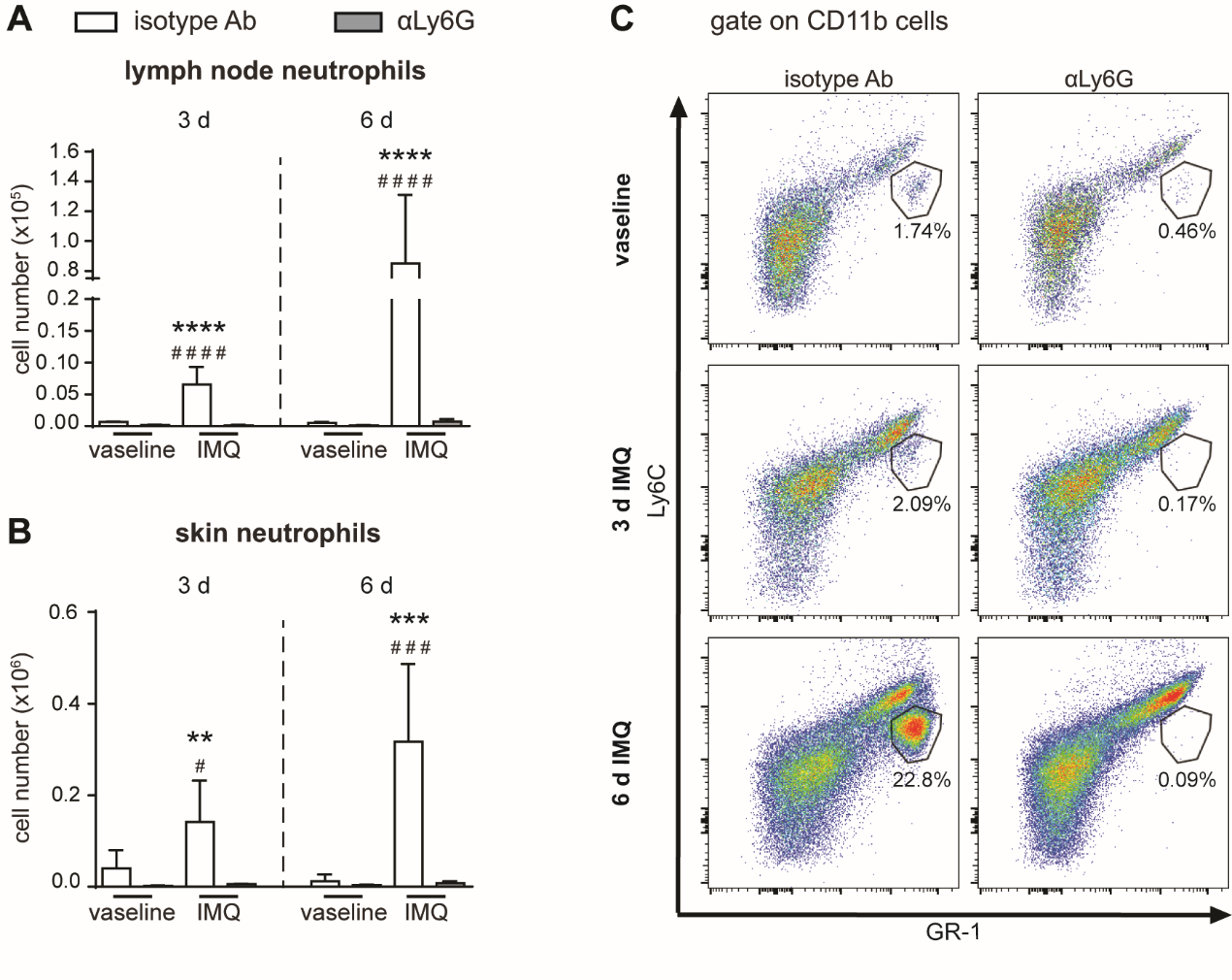


**Supplementary Figure 1. anti-Ly6G antibody (αLy6G) efficiently depletes neutrophils in the lymph nodes and the skin of IMQ-treated mice.** Dorsal skin of mice was topically treated with IMQ containing-cream (Aldara^®^) or vaseline, utilized as control cream, for 3 or 6 consecutive days. To deplete neutrophils, mice were injected i.p. with anti-Ly6G antibody (αLy6G) or the isotype control Ab. (**A**) Draining lymph nodes were collected and analysed by flow cytometry. The total number of neutrophils (CD11b^+^Ly6C^int^GR-1^high^) is reported. (**B**) Total skin (2x2 cm) was digested and analysed by flow cytometry. The total number of neutrophils (CD11b^+^Ly6C^int^GR-1^high^) is reported. (**C**) Representative FACS plots showing the frequencies of CD11b^+^Ly6C^int^GR-1^high^ neutrophils in the lymph nodes of αLy6G- or isotype Ab- treated mice after treatment with IMQ, or vaseline, for 3 or 6 consecutive days. Data are pooled from 2 separate time course experiments and are expressed as means ± SD (*n* = 5 mice per time point). Statistical differences of IMQ-treated vs. vaseline-treated mice (#) and IMQ-treated vs. IMQ-treated mice following neutrophil depletion (*) are reported. #*P* ≤ 0.05; ***P* ≤ 0.01; ###/****P* ≤ 0.001; ####/**** *P* ≤ 0.0001 by 1-way ANOVA with Bonferroni’s post-test.

**
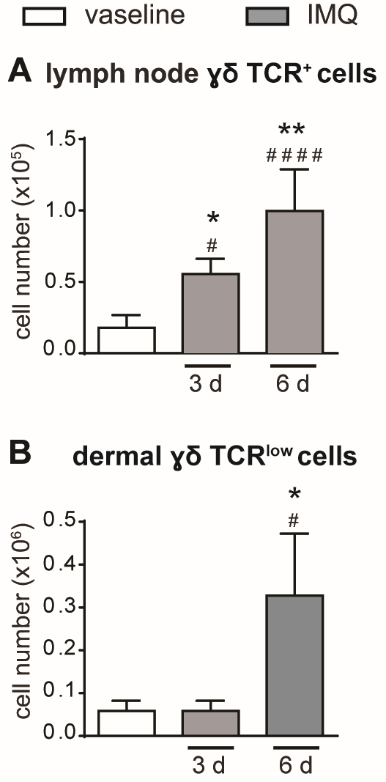
**

**Supplementary Figure 2. Infiltration of γδ T cells in the lymph nodes and skin of mice treated with IMQ for 3 and 6 days.** Dorsal skin of mice was topically treated with vaseline or IMQ-containing cream (Aldara^®^) for 3 or 6 consecutive days. (**A**) Draining lymph nodes were collected and analyzed by flow cytometry. The total number of γδ TCR^+^ cells is reported. (**B**) Total skin (2x2 cm) was digested and analyzed by flow cytometry. The total number of dermal γδ TCR^low^ T cells is reported. Data are pooled from 2 separate time course experiments and are expressed as means ± SD (n = 5 mice). Statistical differences of IMQ-treated vs. vaseline-treated mice (#) and IMQ-treated mice after 3 days vs. IMQ-treated mice after 6 days (*) are reported. #/**P* ≤ 0.05; ***P* ≤ 0.01; #### *P* ≤ 0.0001 by 1-way ANOVA with Bonferroni’s post-test.

**
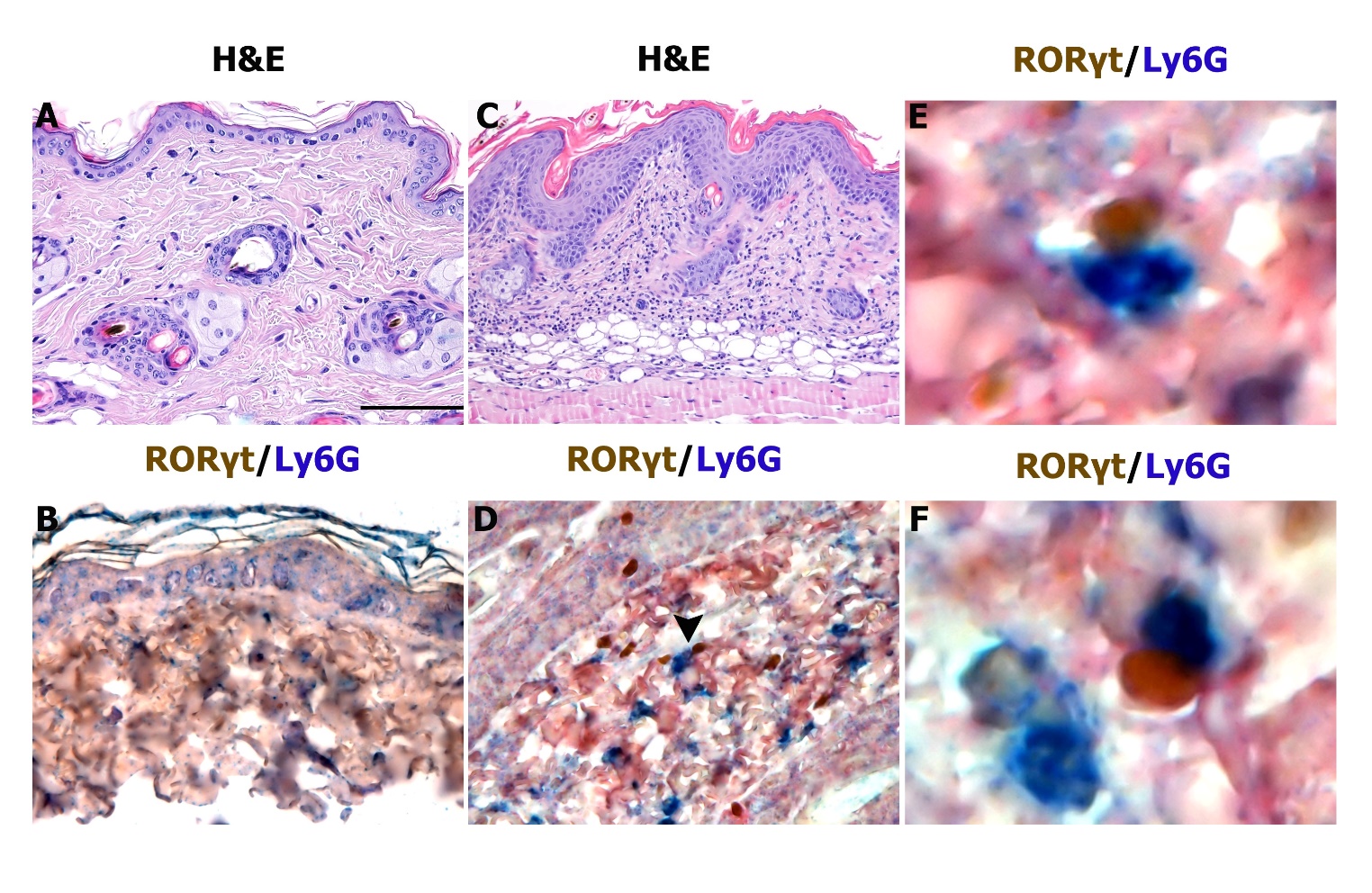
**

**Supplementary Figure 3. γδ T cells are in close contact with neutrophils in the skin of mice treated with IMQ for 6 days.** The Dorsal skin of mice was topically treated with vaseline or IMQ-containing cream (Aldara^®^) for 6 consecutive days. Sections are from mouse formalin-fixed paraffin embedded skin and stained as labelled. Compared to control (A, B) treated skin (C-F) display hyperkeratosis and keratinocytes hyperplasia with dermal immune infiltration (C, D). Arrow in D indicates a γδ T cell in close contact with a neutrophil. The digital magnifications (E, F) illustrate a high-power view of cell contacts. Original magnifications: 100x (C), scale bar 200 microns; 200x (A), scale bar 100 microns; 400x (B, D), scale bar 50 microns; digital resize of 400x photos (E, F), scale bar 17 microns.

**
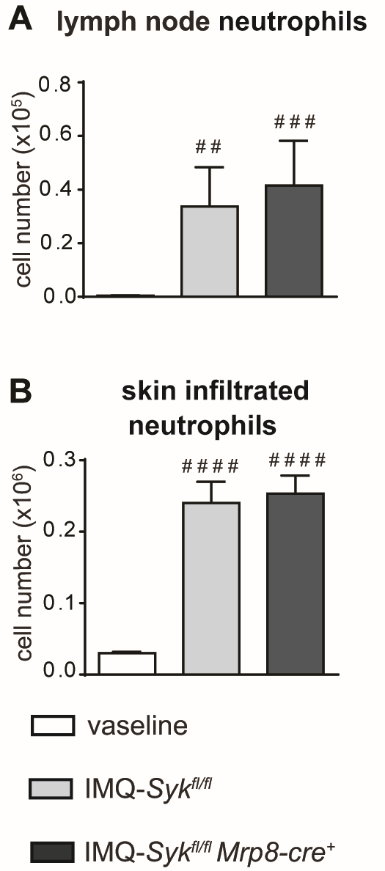
**

**Supplementary Figure 4. Infiltration of neutrophils in the lymph nodes and skin of IMQ-treated *Syk^fl/fl^* and *Syk^fl/fl^Mrp8-cre^+^* mice.** The Dorsal skin of mice was topically treated with vaseline or IMQ-containing cream (Aldara^®^) for 6 consecutive days. (**A**) Draining lymph nodes were collected and analyzed by flow cytometry. The total number of neutrophils is reported. (**B**) Total skin (2x2 cm) was digested and analyzed by flow cytometry. The total number of skin infiltrated neutrophils is reported. Data are pooled from 2 separate time experiments and are expressed as means ± SD (n = 11 mice). Statistical differences of IMQ-treated *Syk^fl/fl^* or *Syk^fl/fl^Mrp8-cre^+^* mice vs. vaseline-treated mice (#) are reported. ##*P* ≤ 0.01; ###*P* ≤ 0.001; ####*P* ≤ 0.0001 by 1-way ANOVA with Bonferroni’s post-test.
